# Clinicopathological characteristics of patients who underwent additional gastrectomy after incomplete endoscopic resection for early gastric cancer

**DOI:** 10.1097/MD.0000000000006172

**Published:** 2017-02-17

**Authors:** Jae Jin Hwang, Dong Ho Lee, Hyuk Yoon, Cheol Min Shin, Young Soo Park, Nayoung Kim

**Affiliations:** a Department of Internal Medicine, Seoul National University College of Medicine, Seoul National University Bundang Hospital, Seongnam, South Korea; b Digestive Disease Center and Research Institute, Department of Internal Medicine, Soonchunhyang University College of Medicine, Soonshunhyang University Bucheon Hospital, Bucheon, South Korea.

**Keywords:** early gastric cancer, endoscopic mucosal resection, endoscopic resection, endoscopic submucosal dissection, gastrectomy

## Abstract

To evaluate the clinicopathological characteristics and factors that lead to residual tumors in patients who underwent additional gastrectomy for incomplete endoscopic resection (ER) for early gastric cancer (EGC).

Between 2003 and 2013, the medical records of patients underwent additional gastrectomy after incomplete ER were retrospectively reviewed. Those diagnosed with the presence of histologic residual tumor in specimens obtained by gastrectomy were assigned to the residual tumor (RT) group (n = 47); those diagnosed with the absence of histologic residual tumor were assigned to the nonresidual tumor (NRT) group (n = 33).

In the multivariate analysis, endoscopic piecemeal resection, *Helicobacter pylori* infection, large tumor size (>2 cm), and both (lateral and vertical) marginal involvement were independent factors of the presence of residual tumor in additional gastrectomy after incomplete resection ER for EGC and the rates of independent factors were significantly higher in the RT group than in the NRT group (*P* < 0.05).

Before ER, preexamination to accurately determine the GC invasion depth and the presence of LN metastasis is very important. During ER, surgeons should attempt to perform en bloc resection and to resect the mucous membrane with adequate safety margins to prevent tumor invasion into the lateral and vertical margins.

## 1 Introduction

Given that health and endoscopic examinations are now commonly performed in clinical practice, the frequency of the diagnosis of early gastric cancer (EGC) among all cases of gastric cancer (GC) has increased. Similarly, in South Korea, an increasing trend of EGC cases has been observed—EGC accounted for 28.6% and 32.8% of all GC cases in 1995 and 1999, respectively.^[1]^ EGC has relatively favorable prognosis, with a 5-year survival rate of ≥90% after radical gastrectomy and a relapse rate of ≤5%.^[2,3]^ Hence, considering the patients’ postoperative quality of life, minimally invasive endoscopic resection (ER) has been widely utilized for cases with EGC localized to the mucous membranes.^[4,5]^

ER is a minimally invasive treatment that was first implemented in 1984 by Tada et al.^[6]^ Since then, ER has been refined, and endoscopic mucosal resection (EMR) and endoscopic submucosal dissection (ESD) techniques have consequently been developed. The indications for ER include depressed-type lesions without the risk of lymph node (LN) metastasis, including those with well-differentiation, but without polypoid lesions <2 cm in diameter or ulcers <1 cm in diameter localized within the mucous membranes.^[7]^ In particular, the limitations of EMR include the increased need for piecemeal resection, possibility of incomplete resection, and likelihood of recurrence; therefore, ESD is being used more frequently. When undergoing ESD, a radical procedure can also be performed since the en bloc and complete resection rates are high, because the submucosal layer is exfoliated under direct observation. In addition, the recent introduction of the extended criteria allows for the procedure to be performed on even larger lesions without restrictions on location.^[8]^ However, the outcomes vary according to the surgeon's competence level and an increased incidence of complications, such as bleeding or perforation, is noted. In addition to the postoperative complications, residual lesions can also be present after ER due to incomplete resection; in such cases, additional gastrectomy would be required.

Recent advancements in endoscopic ultrasonography (EUS) and computed tomography (CT) have increased the accuracy of preoperative LN metastasis diagnosis. Moreover, advancements in endoscopic devices and accessories as well as improved ER competencies have enabled surgeons to perform en bloc resection for even larger lesions. Considering these findings, continuous research is ongoing to expand these indications. However, thus far, only a few studies have examined long-term post-ER follow-up data, and the clinical characteristics of cases with incomplete residual tumors remain unclear. In the present study, the clinicopathological characteristics and factors that lead to residual tumors in patients who underwent additional gastrectomy for incomplete ER for EGC were examined.

## 2 Methods

### 1.1 Patient selection

This study was conducted at Seoul National University Bundang Hospital between January 2003 and December 2013. The medical records of patients underwent additional gastrectomy after incomplete ER were retrospectively reviewed. The patients selected for the study met the following inclusion criteria: age over 18 years; diagnosis of EGC by esophagogastroduodenoscopy (EGD); underwent incomplete ER for EGC, and underwent additional gastrectomy after incomplete ER. The exclusion criteria were as follows: age below 18 years; previous endoscopic submucosal dissection (ESD) or gastric surgery for GC; complete ER for EGC; and underwent additional gastrectomy except incomplete ER.

### 1.2 Endoscopic resection

The ER methods included in this study consisted of EMR (including the injection-and-cut technique, EMR with the cap technique, and EMR by a snare after circumferential precutting with a knife) and ESD. The details of ER have been described previously.^[9]^ Before ER, chromoendoscopy with indigo carmine (0.2% solution) was performed routinely to define the horizontal extent of tumor infiltration. Magnifying endoscopy with narrow-band imaging was selectively performed for delineation of ill-defined margin by chromoendoscopy.

The standard indication for ER was defined as the differentiated histologic type, intramucosal cancer, which has a diameter no larger than 2 cm and no ulceration.^[8]^ The expanded indications for ER were defined as the differentiated histologic type, intramucosal cancer without ulceration irrespective of lesion size; the differentiated histologic type, intramucosal cancer with ulceration and lesion no larger than 3 cm in diameter; and the differentiated histologic type with minute submucosal invasion (invasion <500 μm below the muscularis mucosa) and lesion no larger than 3 cm in diameter.^[4]^

Complete resection was defined as en bloc resection with no cancer cell exposure to any cut end and no lymphovascular invasion.^[10]^ Incomplete resection was defined as resection that did not meet the complete resection criteria. En bloc resection was defined as resection in a one-piece fashion with no residual tumor viewed endoscopically.^[11]^ When the lesion had to be removed in multiple segments, the piecemeal resected specimens were reconstructed as completely as possible.

### 1.3 Histopathologic evaluation

The histopathological diagnoses were based on the Japanese Classification of Gastric Carcinoma.^[11]^ Resected specimens were systematically sectioned at 2 mm intervals, centered on the part of the lesion closest to the margin and the site of deepest invasion. Lesions that remained within the mucosa were classified as mucosal cancers, and those invading the submucosa as submucosal cancers. The histologic differentiation was identified using the World Health Organization criteria,^[12]^ respectively. The cancer location was determined with reference to the Japanese Classification of Gastric Cancer.^[13]^

### 1.4 Study protocol

The enrolled patients were classified into 2 groups. Those diagnosed with the presence of histologic residual tumor in specimens obtained by gastrectomy were assigned to the residual tumor (RT) group; those diagnosed with the absence of histologic residual tumor in specimens obtained by gastrectomy were assigned to the nonresidual tumor (NRT) group. Data on demographics (age, gender, ER method, surgery method, lymphadenectomy method), clinicopathologic characteristics of EGC (location, size, macroscopic finding, histologic diagnosis, histologic grade, histologic type, depth of invasion, invasion of submucosa, marginal involvement, lymphatic invasion, venous invasion, perineural invasion, atrophy of surrounding mucosa, intestinal metaplasia, *Helicobacter pylori* infection), the success or failure of endoscopic en bloc resection, the total number of harvested LNs were recorded. The study protocol was approved by the Ethics Committee of Seoul National University Bundang Hospital (IRB number: L-2014-1020).

### 1.5 Statistical analysis

The statistical analysis was performed using the Predictive Analytics Software (PASW) 20.0 version for Windows package (SPSS, Inc., IBM, Chicago, IL). The mean ± standard deviations of the quantitative variables were calculated. The Student *t* test was used to evaluate the continuous variables, and the Chi-square test and Fisher exact test were utilized to assess the noncontinuous variables. Additionally, univariate and multivariate analyses were conducted to evaluate the independent factors associated with presence of residual tumor in additional gastrectomy after incomplete resection ER for EGC. A *P*-value of less than 0.05 was defined as having clinical significance.

## 2 Results

### 2.1 Comparison of the clinicopathologic characteristics of RT and NRT group

**Figure 1:**
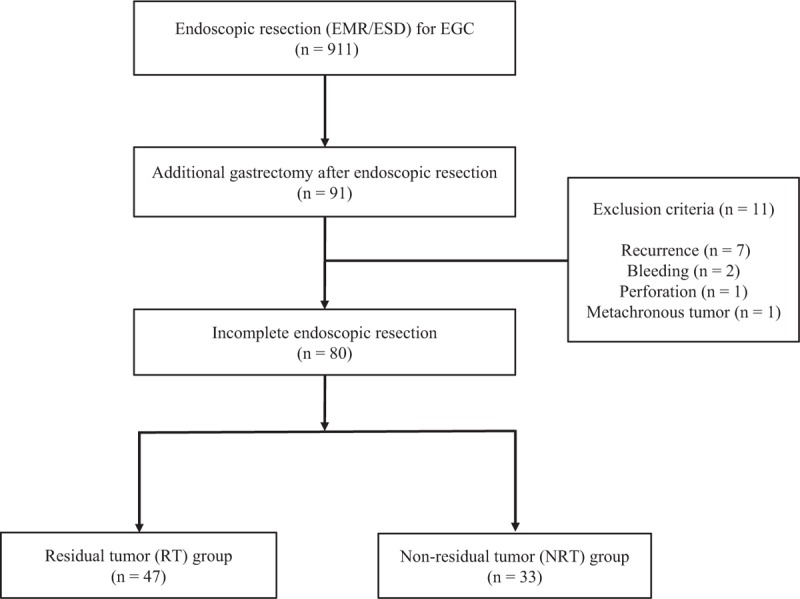
A schematic diagram of the study. EGD = early gastric cancer, EMR = endoscopic mucosal resection, ESD = endoscopic submucosal dissection.

A schematic diagram of the study is provided in Fig. 1. Between 2003 and 2013, 911 patients diagnosed with EGC were underwent ER (EMR/ESD). Of those, 91 patients underwent additional gastrectomy after ER. Of the 91 patients, 11 were excluded from the study because of tumor recurrence (7 patients), bleeding (2 patients), perforation (1 patients), and metachronous (1 patient). Among them, a total 80 patients underwent additional gastrectomy because of incomplete ER. Of those, 47 patients who diagnosed with the presence of histologic residual tumor in specimens obtained by additional gastrectomy were assigned to the residual tumor (RT) group, 33 patients who diagnosed with the absence of histologic residual tumor in specimens obtained by additional gastrectomy were assigned to the nonresidual tumor (NRT) group. The enrolled patients’ baseline demographic and clinical characteristics are provided in Table 1. The average ages of the RT and NRT groups were 67.0 ± 10.7 and 67.8 ± 10.1 years (*P* = 0.727), respectively. The rates of ESD was significantly higher in the NRT group than in the RT group (63.6% vs 31.9%, *P* = 0.005). The *H pylori* infection rates was significantly higher in the RT group than in the NRT group (42.6% vs 18.2%, *P* = 0.021). The rates of mixed macroscopic finding was significantly higher in the RT group than in the NRT group (23.4% vs 9.1%, *P* = 0.041). The rate of tumor size over 2 cm was significantly higher in the RT group than in the NRT group (44.7% vs 27.2%, *P* < 0.001). The rates of endoscopic en bloc resection was significantly lower in the RT group than in the NRT group (61.7% vs 84.8%, *P* = 0.024). The rates of submucosal invasion was significantly higher in the RT group than in the NRT group (76.5% vs 24.2%, *P* = 0.008). The rates of both (lateral and vertical) margin involvement was significantly higher in the RT group than in the NRT group (59.5% vs 15.1%, *P* = 0.027). There were no statistical differences in the gender distribution, atrophy of surrounding mucosa, intestinal metaplasia, tumor location, histology, lymphatic invasion, venous invasion, perineural invasion, surgery method, lymphadenectomy, and total number of harvested LNs between the 2 groups (Table 1).

**Table 1:**
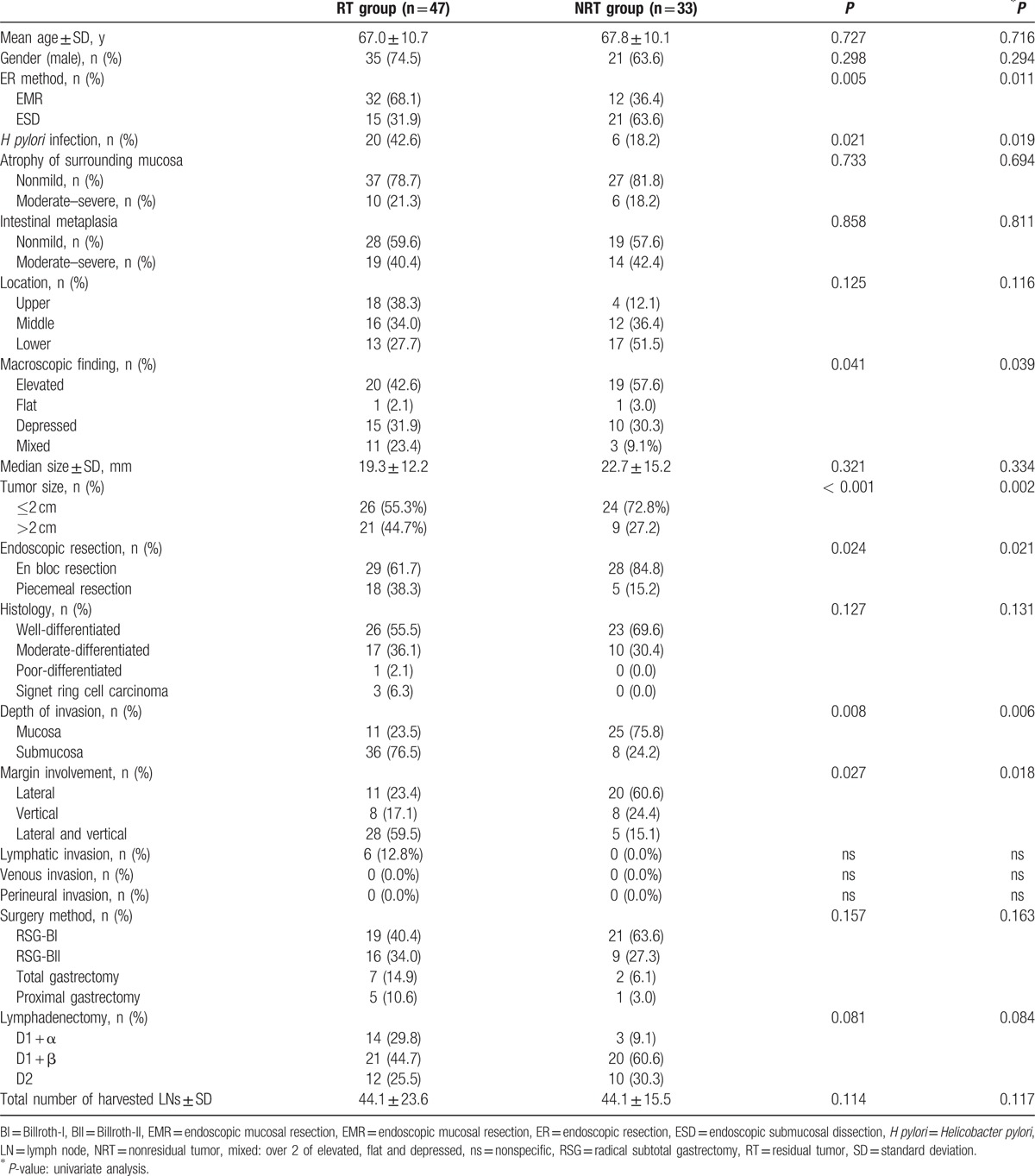
Clinicopathologic characteristics and univariate analysis of the risk factors associated with presence of residual tumor of the patients who underwent additional gastrectomy after incomplete resection ER for early gastric cancer.

### 2.2 Clinical factors influencing the presence of residual tumor

To evaluate the clinical factors influencing the presence of residual tumor in additional gastrectomy after incomplete resection ER for EGC, univariate analyses were performed, which are listed in Table 1. *H pylori* infection, mixed macroscopic finding, large tumor size (>2 cm), endoscopic piecemeal resection, submucosal invasion, and both (lateral and vertical) marginal involvement were associated with the presence of residual tumor in additional gastrectomy after incomplete resection ER for EGC (*P* < 0.05). The multivariate analysis revealed that endoscopic piecemeal resection (odds ratio [OR]: 5.02, 95% confidence interval [CI]: 3.78–6.24, *P* = 0.048), *H pylori* infection (OR: 1.89, 95% CI: 1.36–2.42, *P* = 0.041), large tumor size (>2 cm, OR: 2.96, 95% CI: 2.37–3.55, *P* = 0.022), and both (lateral and vertical) marginal involvement (OR: 4.96, 95% CI: 4.13–5.79, *P* = 0.011) were independent factors, predictive of the presence of residual tumor in additional gastrectomy after incomplete resection ER for EGC (Table 2).

**Table 2:**
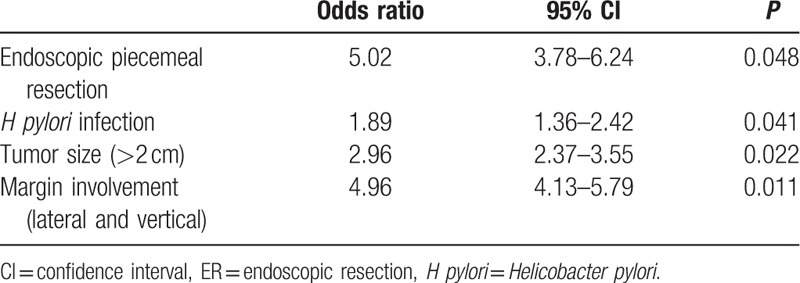
Multivariate analysis of the risk factors associated with presence of residual tumor in additional gastrectomy after incomplete resection ER for early gastric cancer.

### 2.3 Comparison of the rates of endoscopic resection and margin involvement between RT and NRT group according to ER method

Table 3 shows the comparison of the rates of ER and margin involvement between RT and NRT group according to ER method. The rates of en bloc resection in the patients with ESD method were significantly higher than that in the patients with EMR method in the both (RT and NRT) groups (86.7% vs 50.0%, 58.3% vs 100.0%, *P* < 0.05). The rates of margin involvement in the patients with ESD method were significantly lower than that in the patients with EMR method in the both (RT and NRT) groups (*P* < 0.05, Table 3).

**Table 3:**
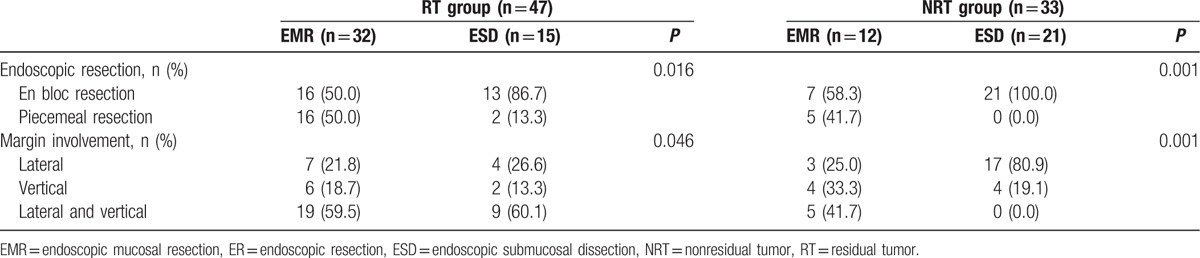
Comparison of the rates of endoscopic resection and margin involvement between RT and NRT group according to ER method.

### 2.4 Relationship between positive margins, depth of invasion with residual tumors and lymph node metastasis

Table 4 shows the relationship between positive margins, depth of invasion with residual tumors and LN metastasis. The rate of residual tumor was 35.4% (11/31) in the positive lateral margin, 50.0% (8/16) in the positive vertical margin, and 84.8% (28/33) in the positive lateral and vertical margin. There was 1 case with LN metastasis in the positive lateral margin and 5 cases in the positive lateral and vertical margin (Table 5). The rate of residual tumor was 30.5% (11/36) in the mucosal invasion and 81.8% (36/44) in the submucosal invasion. All cases with LN metastasis were submucosal tumor (Table 4).

**Table 4:**
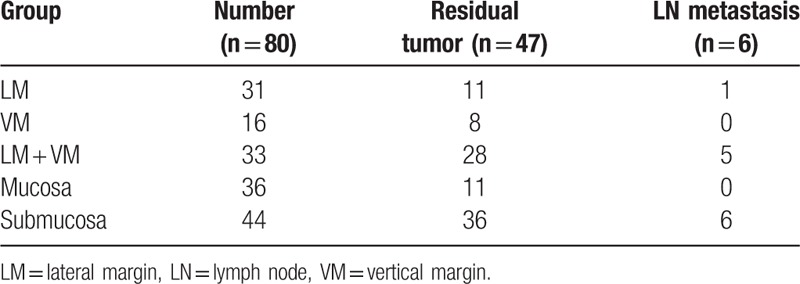
Relationship between positive margins, depth of invasion with residual tumors and lymph node metastasis.

**Table 5:**
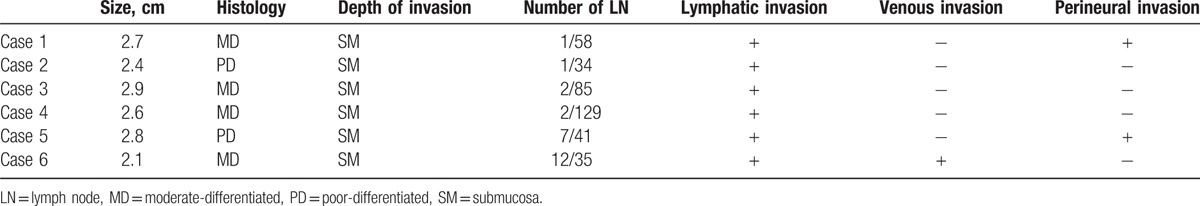
Details of 6 patients with LN metastasis.

### 2.5 Cases of lymph node metastasis

Table 5 shows the characteristics of 6 patients with LN metastasis. There were 6 cases of LN metastasis. The sizes of tumors were over 2 cm in all cases. The histological diagnosis were moderately differentiated for Case 1, 3, 4, and 6, and poorly differentiated in Case 2 and 5. The depths of invasion were submucosal invasion in all cases. The number of LN metastasis were 1, 1, 2, 2, 7, and 12. Case 1 and 5 had perineural invasions and Case 6 had venous invasions (Table 5).

## 3 Discussion

Due to advancements in endoscopic technology, the use of ER procedures in the treatment of EGC is increasing. ER was first performed for the treatment of EGC in Japan in the late 1980s^[14]^ and has advanced substantially since then. In Japan, EMR is performed in approximately 50% of the patients with EGC,^[15]^ and its use is increasing in South Korea as well. An ESD method that involves direct exfoliation of the submucosal areas with a knife was introduced to allow for en bloc resection of larger lesions.^[8,16]^ The en bloc and complete resection rates of ESD are reportedly both 92.8%, which is higher than those of EMR (43.4% and 24.6%, respectively).^[8]^ However, the disadvantages of this ESD method include the high incidence of complications and change in the outcomes according to the surgical techniques employed and the surgeon's competence level; furthermore, in ESD, additional gastrectomy may be required in cases with incomplete resection. Therefore, to reduce the frequency of additional gastrectomy after ER, studies on the clinicopathological characteristics and factors that lead to residual tumors in patients who undergo additional gastrectomy due to incomplete ER for EGC are needed.

Korenaga et al^[17]^ examined 11 patients who underwent additional gastrectomy after incomplete EMR, and stated that the possibility of residual tumor was high when the cases involved a submucosal tumor or tumor invasions in the resection margin. Moreover, Nagano et al^[18]^ reported that the risk of residual tumor was high and the risk of LN metastasis was present after EMR in cases with submucosal tumors or tumor invasions in the vertical margin; these cases would require radical resection and lymphadenectomy. In contrast, among cases where the lesions were limited to the mucous membranes and tumor invasion was limited to the lateral margin only, the residual tumor was found only in 5.8% (18/309) of cases, without any LN metastasis; these cases would require close follow-up and additional endoscopic treatments. In the present study, the frequency of additional gastrectomy due to incomplete resection after ER for EGC was 8.7% (80/911), and 58.7% (47/80) of those patients had residual tumors, similar to the results of Korenaga et al.^[17]^ As in previous studies, the present study showed a distinctively high possibility of residual tumors in patients with submucosal tumors or tumor invasion to the resection margin, particularly among cases with invasion to both lateral and vertical margins. Since ER used a coagulating device to resect the tissues, the resection surface was damaged by electricity, which prevented accurate judgments; moreover, when the invasions occurred in both margins, instead of just the lateral or vertical margin, the possibility of residual tumors was higher as the residual tumors would be deeper and wider.

In the present study, one factor associated with the risk of residual tumor was the implementation of an en bloc resection. This finding is believed to be associated with the implementation of piecemeal resections for lesions wherein complete histological evaluation was impossible, reconstruction of tissues that underwent piecemeal resection was difficult, and various resection surfaces were damaged by electricity—all these factors make accurate determinations difficult. Song et al^[19]^ reported that piecemeal resection during EMR led to either residual GC tissue or tumor recurrence, whereas Ono et al^[20]^ stated that the implementation of piecemeal resection was the most important factor in recurrence after EMR. Recent studies reported that the emergence of ESD, which is more advanced than EMR, has made en bloc resection easier, which can consequently reduce recurrence. Moreover, when ESD is used, en bloc resection makes it easier to evaluate the resection completeness, and thus reduce recurrence.^[8,21]^ In our study, the ESD method showed higher rates of en bloc resection and lower rates of margin involvement than the EMR method as previous studies.^[19,20]^ Therefore, to reduce the possibility of residual tumors, the implementation of en bloc resection, whenever possible, is believed to be important.

Ohnita et al^[22]^ indicated that when lesions are >3 cm in diameter or located in the upper portion of the stomach with ulcers, precautions should be taken during ESD with respect to preventing perforation and ensuring complete treatment. In the present study, in cases with tumors >2 cm in size or with mixed macroscopic findings that were impossible to visually differentiate via endoscopy, the possibility of residual tumors was high. One of the reasons for this finding may be associated with the difficulties in defining the resection boundaries, considering that ER boundaries would increase with increase in lesion size, and may also be related to the difficulties in verifying the clear-cut boundaries when mixed macroscopic findings are observed on endoscopy.

In the present study, the patients with residual tumors showed a high *H pylori* infection rate. *H pylori* infection is a known risk factor of GC development,^[23,24]^ and many studies reported that it is associated with atrophic changes in mucosa and intestinal metaplasia, which are indicative of gastric carcinogenesis.^[25–27]^ When a *H pylori* infection is present, the possibility of accompanying atrophic changes in the mucosa and intestinal metaplasia is high in lesions and surrounding tissues; these factors reportedly cause difficulties in the accurate confirmation of lesions and the determination of resection areas, along with increased difficulty performing ER. Therefore, during the endoscopic diagnosis of EGC, the presence of *H pylori* infection must be tested; if *H pylori* infection is detected, eradication therapy might be performed to lower the possibility of residual tumor.

The frequency of LN metastasis in EGC with confirmed submucosal layer invasion is known to be 20%.^[28]^ According to Shimada et al,^[29]^ the frequency of LN metastasis in lesions accompanied by submucosal layer invasion was 19.8%. Ishikawa et al^[30]^ reported an association between the tumor depth of the wall invasion and LN metastasis; of 15 cases of differentiated EGC without ulcers accompanied by submucosal layer invasion <500 μm in depth, LN metastasis was confirmed in 2 (13%). In contrast, Gotoda et al^[4]^ reported no risk of LN metastasis in cases with differentiated adenocarcinoma, lesions <3 cm in size, no lymphatic vessels or vascular invasion, and submucosal tumor invasion depth of <500 μm. All the cases with LN metastases observed in the present study (13.6% [6/44]) had a lesion size of <3 cm and were confirmed to have submucosal tumors, which was similar to the results reported by Ishikawa et al.^[30]^ Moreover, of the 6 cases with confirmed LN metastasis, 5 (83.3%) showed both lateral and vertical margin invasion. Therefore, as the risk of LN metastasis is high in cases with deep invasion after ER or in cases with both lateral and vertical margin invasions on post-ER pathological examination, additional gastrectomy should be considered in such cases.

Before treatment, elucidation of the tumor invasion depth and confirmation of LN metastasis are important to determine the appropriate therapeutic method for GC. Tsendsuren et al^[31]^ reported that during the preoperative diagnosis of GC stage via EUS, the accuracies of T1, T2, T3, and T4 staging were 68.3%, 93.3%, 60%, and 100%, respectively, whereas the diagnostic accuracy for LN metastasis was 66%. Chen et al^[32]^ also reported that, by using EUS, the T-stage had a diagnostic accuracy of 88% and the N-stage had accuracy, sensitivity, and specificity of 79%, 79%, and 80%, respectively. Moreover, some reports indicated that the diagnostic accuracy of multidetector computed tomography (MDCT) for LN metastasis ranges from 78% to 86%.^[33,34]^ Furthermore, when ER is performed as a treatment for GC, the pathological findings from the resected tissues during ER are very important to determine whether additional gastrectomy is needed.

The present study had a limitation. Since it was a retrospective study based on patients’ medical records, the prognoses of the patients who underwent additional gastrectomy after ER were not investigated.

The present study investigated the clinicopathological characteristics and factors that lead to residual tumors in patients who underwent additional gastrectomy for incomplete ER for EGC. The possibility of residual tumor and the need for additional gastrectomy were high in cases wherein piecemeal resection was performed during ER, lesions were >2 cm in size, *H pylori* infection was present, or tumor invasions developed at both the lateral and vertical margins. Moreover, the prevalence of LN metastasis was high in cases with submucosal invasion and tumor invasions to both the lateral and vertical margins. The ESD method showed higher rates of en bloc resection and lower rates of margin involvement than the EMR method. Therefore, when EGC is observed, a thorough endoscopic examination along with EUS and MDCT should be used to accurately determine the GC invasion depth and the presence of LN metastasis. Based on these findings, the appropriate treatment should be chosen. In cases of *H pylori* infection, eradication treatment might be administered. Moreover, surgeons should attempt to perform en bloc resection when performing ER and attempt to resect the mucous membrane with adequate safety margins to prevent tumor invasion into the lateral and vertical margins. The ESD method is more effective and has higher rates of en bloc resection and lower rates of margin involvement than the EMR method as an endoscopic treatment for the complete resection to prevent tumor invasion into the lateral and vertical margins. Furthermore, effort should be made to ensure timely and accurate decisions about whether additional gastrectomy is needed using the pathological findings of the resected tissues after ER, to ensure that there is no delay in surgery.
